# Crystal structure of [(*E*)-({2-[3-(2-{(1*E*)-[(carbamo­thioyl­amino)­imino]­meth­yl}phen­oxy)prop­oxy]phen­yl}methyl­idene)amino]­thio­urea with an unknown solvate

**DOI:** 10.1107/S2056989015012074

**Published:** 2015-06-30

**Authors:** Joel T. Mague, Shaaban K. Mohamed, Mehmet Akkurt, Sabry H. H. Younes, Mustafa R. Albayati

**Affiliations:** a Department of Chemistry, Tulane University, New Orleans, LA 70118, USA; b Faculty of Science & Engineering, School of Healthcare Science, Manchester Metropolitan University, Manchester M1 5GD, England; c Chemistry Department, Faculty of Science, Minia University, 61519 El-Minia, Egypt; d Department of Physics, Faculty of Sciences, Erciyes University, 38039 Kayseri, Turkey; e Chemistry Department, Faculty of Science, Sohag University, 82524 Sohag, Egypt; f Kirkuk University, College of Education, Department of Chemistry, Kirkuk, Iraq

**Keywords:** crystal structure, bis-thio­semicarbazones, biological activity, SQUEEZE

## Abstract

The title mol­ecule, C_19_H_22_N_6_O_2_S_2_, has crystallographically imposed *C*_2_ symmetry, with the central C atom lying on the rotation axis. The O—C—C—C torsion angle for the central chain is −59.22 (16)° and the dihedral angle between the planes of the benzene rings is 75.20 (7)°. In the crystal, N—H⋯O and N—H⋯S inter­actions link the mol­ecules, forming a three-dimensional network encompassing channels running parallel to the *c* axis, which account for about 20% of the unit-cell volume. The contribution to the scattering from the highly disordered solvent mol­ecules in these channels was removed with the SQUEEZE routine [Spek (2015). *Acta Cryst*. C**71**, 9–18] in *PLATON*. The stated crystal data for *M*_r_, μ *etc.* do not take these into account.

## Related literature   

For the various biological activities of bis-thio­semicarbazones, see: Singh *et al.* (2001 ▸); Offiong & Martelli (1997 ▸). For general synthesis and assessment of the pharmaceutical properties of thio­semicarbazone scaffold compounds, see: Greenbaum *et al.* (2004 ▸); Finch *et al.* (1999 ▸); Wilson *et al.* (1974 ▸); Du *et al.* (2002 ▸); Desai *et al.* (1984 ▸); Shucla *et al.* (1984 ▸); Vrdoljak *et al.* (2010 ▸); Belicchi-Ferrari *et al.* (2010 ▸); Marzano *et al.* (2009 ▸). For use of the SQUEEZE routine in *PLATON* to remove the contribution of disordered solvents, see: Spek (2009 ▸, 2015 ▸).
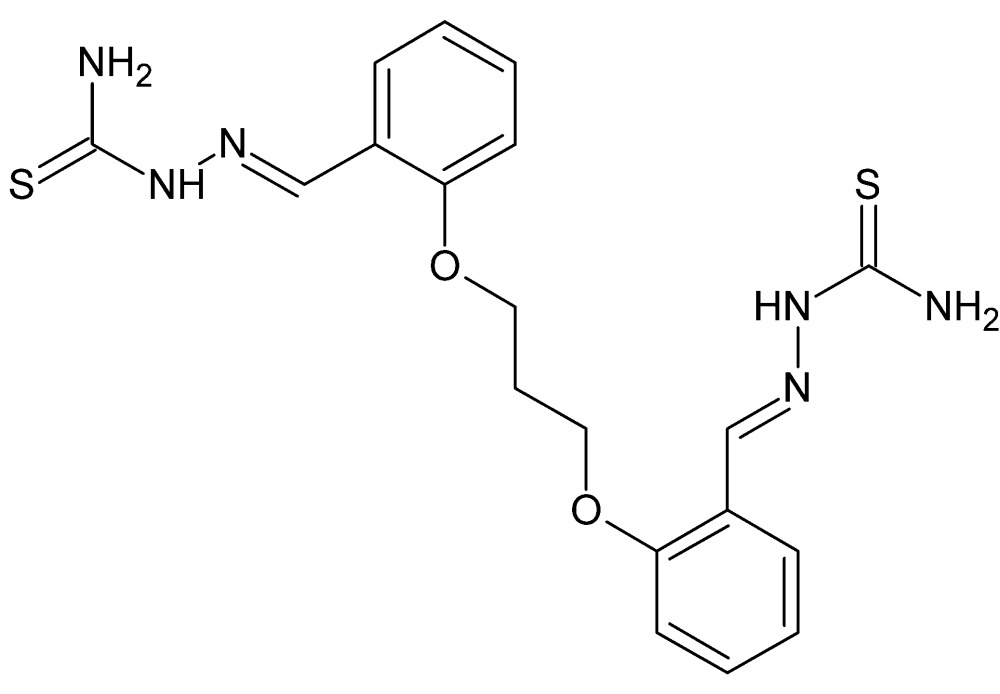


## Experimental   

### Crystal data  

C_19_H_22_N_6_O_2_S_2_
*M* *_r_* = 430.55
Monoclinic, 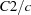

*a* = 19.3941 (5) Å
*b* = 12.7110 (3) Å
*c* = 10.1450 (3) Å
β = 103.306 (2)°
*V* = 2433.79 (11) Å^3^
*Z* = 4
Cu *K*α radiation
μ = 2.19 mm^−1^
*T* = 150 K
0.44 × 0.23 × 0.05 mm

### Data collection  

Bruker D8 VENTURE PHOTON 100 CMOS diffractometer
Absorption correction: multi-scan (*SADABS*; Bruker, 2014 ▸) *T*_min_ = 0.71, *T*_max_ = 0.91
8997 measured reflections
2365 independent reflections
1886 reflections with *I* > 2σ(*I*)
*R*_int_ = 0.042

### Refinement  

*R*[*F* ^2^ > 2σ(*F* ^2^)] = 0.040
*wR*(*F* ^2^) = 0.112
*S* = 1.06
2365 reflections
135 parameters
1 restraint
H atoms treated by a mixture of independent and constrained refinement
Δρ_max_ = 0.24 e Å^−3^
Δρ_min_ = −0.22 e Å^−3^

### 

Data collection: *APEX2* (Bruker, 2014 ▸); cell refinement: *SAINT* (Bruker, 2014 ▸); data reduction: *SAINT*; program(s) used to solve structure: *SHELXT* (Sheldrick, 2015*a* ▸); program(s) used to refine structure: *SHELXL2014* (Sheldrick, 2015*b* ▸); molecular graphics: *DIAMOND* (Brandenburg & Putz, 2012 ▸) and *ORTEP-3 for Windows* (Farrugia, 2012 ▸); software used to prepare material for publication: *SHELXTL* (Sheldrick, 2008 ▸) and *WinGX* (Farrugia, 2012 ▸).

## Supplementary Material

Crystal structure: contains datablock(s) global, I. DOI: 10.1107/S2056989015012074/hb7453sup1.cif

Structure factors: contains datablock(s) I. DOI: 10.1107/S2056989015012074/hb7453Isup2.hkl

Supporting information file. DOI: 10.1107/S2056989015012074/hb7453Isup3.cml

**Fig. 1.**
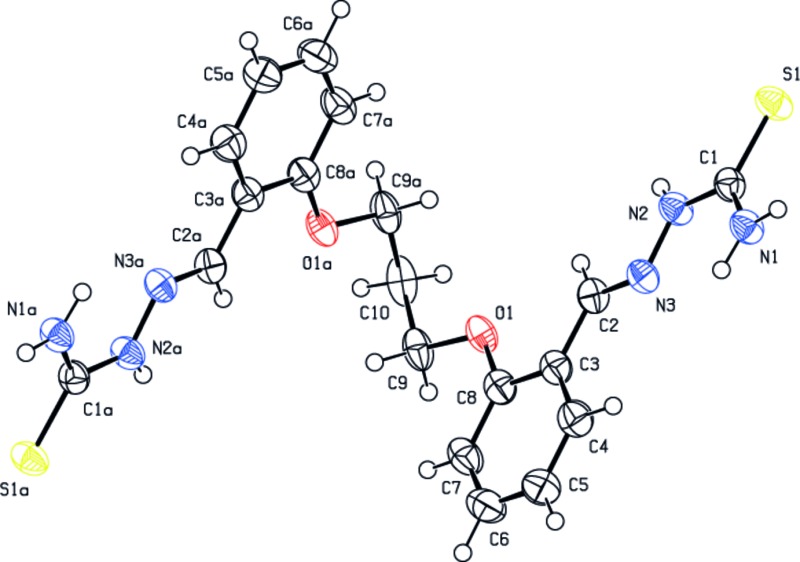
The title molecule with labeling scheme and 50% probability ellipsoids. Atoms with the suffix a are related to their counterparts by the crystallographic twofold axis passing through C10.

**Fig. 2.**
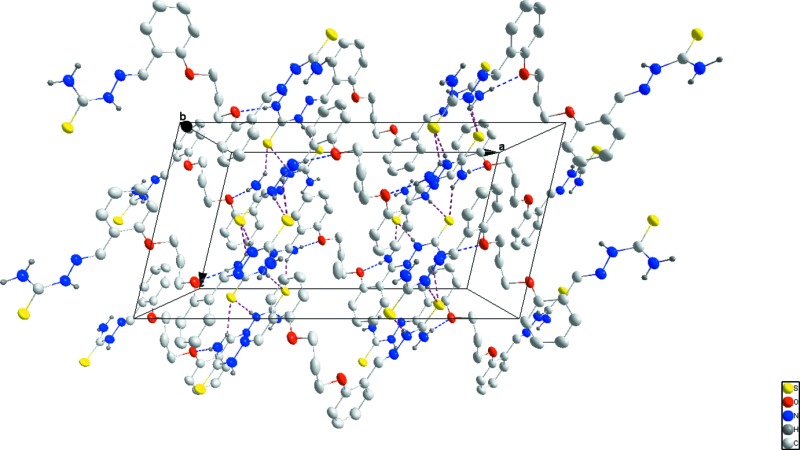
Packing viewed down the b axis. N—H···O and N—H···S hydrogen bonds are shown, respectively, as blue and purple dotted lines.

**Fig. 3.**
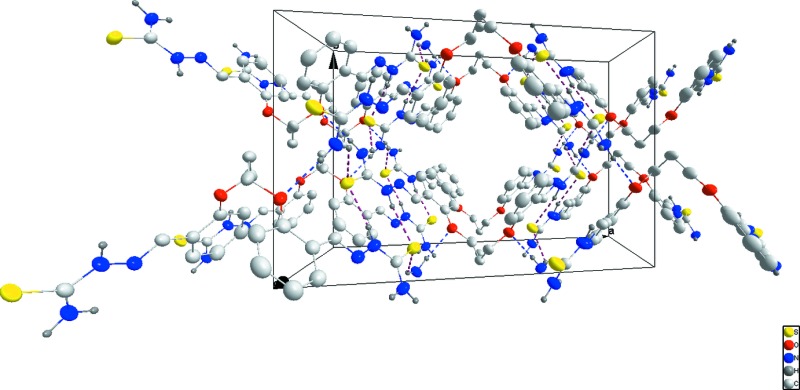
Packing viewed down the the c axis showing the one-dimensonal channels.

. DOI: 10.1107/S2056989015012074/hb7453fig1.tif
The title mol­ecule with labeling scheme and 50% probability ellipsoids. Atoms with the suffix a are related to their counterparts by the crystallographic twofold axis passing through C10.

b . DOI: 10.1107/S2056989015012074/hb7453fig2.tif
Packing viewed down the *b* axis. N—H⋯O and N—H⋯S hydrogen bonds are shown, respectively, as blue and purple dotted lines.

c . DOI: 10.1107/S2056989015012074/hb7453fig3.tif
Packing viewed down the the *c* axis showing the one-dimensonal channels.

CCDC reference: 1408451

Additional supporting information:  crystallographic information; 3D view; checkCIF report

## Figures and Tables

**Table 1:** Hydrogen-bond geometry (, )

*D*H*A*	*D*H	H*A*	*D* *A*	*D*H*A*
N1H1*A*N3	0.91	2.27	2.631(2)	103
N1H1*A*S1^i^	0.91	2.64	3.3393(16)	135
N1H1*B*O1^ii^	0.91	2.20	3.1046(19)	176
N2H2*A*S1^iii^	0.91	2.49	3.3909(16)	171

Symmetry codes: (i) 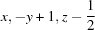; (ii) 
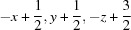
; (iii) 
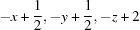
.

## supplementary crystallographic information

### S1. Comment

Currently, bis-thiosemicarbazones is considerable interest in their biological activity (Singh *et al.*, 2001; Offiong & Martelli, 1997) and have been known for over 50 years. Thiosemicarbazones have been reported to exhibit antivirals and as anticancer therapeutics, as well as for their parasiticidal action against Plasmodium falciparum and Trypanasoma cruzi which are the causative agents of malarya and Chagas' disease, respectively (Greenbaum *et al.*, 2004; Finch *et al.*, 1999; Wilson *et al.*, 1974; Du *et al.*, 2002). In addition, in the last few years there has been a growing attention towards thiosemicarbazones related to their range of biological properties, as antituberculosis activity (Desai *et al.*, 1984; Shucla *et al.*, 1984), antitumor (Vrdoljak *et al.*, 2010), antiproliferative (Belicchi-Ferrari *et al.*, 2010), and anticancer agents (Marzano *et al.*, 2009). Such facts inspired us to synthesis and study the crystal structure determination of the title compound.

The title molecule has crystallographically imposed C_2_ symmetry (Fig. 1). The dihedral angle between the planes of the benzene rings is 75.20 (7)°. Significant N1—H1B···O1^i^ (i: 1.5 - *x*, -1/2 + *y*, 1/2 - *z*) hydrogen bonds are formed in the crystal as well as weaker N2—H2A···S1^ii^ (ii: 1.5 - *x*, 1.5 - *y*, -*z*) and N1—H1A···S1^iii^ (iii: *x* 1 - *y*, 1/2 + *z*) interactions (Fig. 2). These lead to the formation of channels running parallel to the *c* axis (Fig. 3).

### S2. Experimental

A mixture of 0.5 mmol (142 mg) of 2,2'-[ethane-1,2-diylbis(oxy)]dibenzaldehyde and 1 mmol (91 mg) of thiosemicarbazide in ethanol (10 ml) was heated under reflux for 4 h in the presence of a catalytic amount of acetic acid. After cooling, the reaction mixture was poured into an ice-water. The resulting solid product was then filtered off, washed with water, dried and crystallized from dimethylformamide to afford the title compound. Mp 488 K.

### S3. Refinement

The H-atom (H10A) attached to C10 was located from a difference Fourier map and refined with restraint C—–H = 0.99 (2) Å using a riding model, with *U*_iso_(H) = 1.2 *U*_eq_(C). The other H-atoms attached to carbon were placed in calculated positions (C—H = 0.95 - 0.99 Å) while those attached to nitrogen were placed in locations derived from a difference map and their parameters adjusted to give N—H = 0.91 Å. All were included as riding contributions with isotropic displacement parameters 1.2 times those of the attached atoms. A region of density amounting to the scattering from approximately 1.5 carbon atoms, apparently disordered about the twofold axis and well removed from the main molecule was removed with *PLATON SQUEEZE* (Spek, 2009) after it proved impossible to identify it with any reasonable solvent or byproduct molecule.

### Crystal data

**Table:**

C_19_H_22_N_6_O_2_S_2_	*F*(000) = 904
*M**_r_* = 430.55	*D*_x_ = 1.175 Mg m^−^^3^
Monoclinic, *C*2/*c*	Cu *K*α radiation, λ = 1.54178 Å
Hall symbol: -C 2yc	Cell parameters from 5935 reflections
*a* = 19.3941 (5) Å	θ = 4.2–72.3°
*b* = 12.7110 (3) Å	µ = 2.19 mm^−^^1^
*c* = 10.1450 (3) Å	*T* = 150 K
β = 103.306 (2)°	Plate, colourless
*V* = 2433.79 (11) Å^3^	0.44 × 0.23 × 0.05 mm
*Z* = 4	

### Data collection

**Table:**

Bruker D8 VENTURE PHOTON 100 CMOS diffractometer	2365 independent reflections
Radiation source: INCOATEC IµS micro–focus source	1886 reflections with *I* > 2σ(*I*)
Mirror monochromator	*R*_int_ = 0.042
Detector resolution: 10.4167 pixels mm^-1^	θ_max_ = 72.4°, θ_min_ = 4.7°
ω scans	*h* = −23→21
Absorption correction: multi-scan (*SADABS*; Bruker, 2014)	*k* = −15→15
*T*_min_ = 0.71, *T*_max_ = 0.91	*l* = −12→11
8997 measured reflections	

### Refinement

**Table:**

Refinement on *F*^2^	1 restraint
Least-squares matrix: full	Hydrogen site location: mixed
*R*[*F*^2^ > 2σ(*F*^2^)] = 0.040	H atoms treated by a mixture of independent and constrained refinement
*wR*(*F*^2^) = 0.112	*w* = 1/[σ^2^(*F*_o_^2^) + (0.0642*P*)^2^ + 0.5713*P*] where *P* = (*F*_o_^2^ + 2*F*_c_^2^)/3
*S* = 1.06	(Δ/σ)_max_ = 0.001
2365 reflections	Δρ_max_ = 0.24 e Å^−^^3^
135 parameters	Δρ_min_ = −0.22 e Å^−^^3^

### Special details

**Table:**

Geometry. Bond distances, angles *etc*. have been calculated using the rounded fractional coordinates. All su's are estimated from the variances of the (full) variance-covariance matrix. The cell e.s.d.'s are taken into account in the estimation of distances, angles and torsion angles
Refinement. Refinement on *F*^2^ for ALL reflections except those flagged by the user for potential systematic errors. Weighted *R*-factors *wR* and all goodnesses of fit *S* are based on *F*^2^, conventional *R*-factors *R* are based on *F*, with *F* set to zero for negative *F*^2^. The observed criterion of *F*^2^ > σ(*F*^2^) is used only for calculating -*R*-factor-obs *etc*. and is not relevant to the choice of reflections for refinement. *R*-factors based on *F*^2^ are statistically about twice as large as those based on *F*, and *R*-factors based on ALL data will be even larger.

### Fractional atomic coordinates and isotropic or equivalent isotropic displacement parameters (Å^2^)

**Table:**

	*x*	*y*	*z*	*U*_iso_*/*U*_eq_	
S1	0.16947 (3)	0.37583 (4)	1.00065 (5)	0.0410 (2)	
O1	0.41803 (6)	0.15260 (9)	0.63827 (14)	0.0374 (4)	
N1	0.18026 (8)	0.48300 (11)	0.78226 (16)	0.0378 (5)	
N2	0.24940 (8)	0.33630 (12)	0.82840 (15)	0.0362 (5)	
N3	0.27239 (8)	0.35523 (11)	0.71219 (16)	0.0355 (4)	
C1	0.20151 (9)	0.40093 (13)	0.86192 (18)	0.0338 (5)	
C2	0.31703 (9)	0.28864 (13)	0.68644 (18)	0.0343 (5)	
C3	0.34370 (10)	0.29762 (13)	0.56355 (19)	0.0356 (5)	
C4	0.31914 (10)	0.37535 (16)	0.4677 (2)	0.0437 (6)	
C5	0.34321 (11)	0.38278 (17)	0.3499 (2)	0.0493 (7)	
C6	0.39290 (12)	0.31119 (18)	0.3267 (2)	0.0499 (7)	
C7	0.41864 (11)	0.23341 (16)	0.4199 (2)	0.0443 (6)	
C8	0.39431 (10)	0.22645 (13)	0.53912 (19)	0.0358 (5)	
C9	0.47639 (10)	0.08725 (14)	0.6228 (2)	0.0421 (6)	
C10	0.50000	0.0236 (2)	0.75000	0.0468 (9)	
H1A	0.19900	0.49480	0.70920	0.0450*	
H1B	0.15310	0.53380	0.80830	0.0450*	
H2	0.33300	0.23270	0.74800	0.0410*	
H2A	0.26650	0.27530	0.87040	0.0430*	
H4	0.28500	0.42450	0.48350	0.0520*	
H5	0.32590	0.43640	0.28560	0.0590*	
H6	0.40940	0.31570	0.24570	0.0600*	
H7	0.45280	0.18470	0.40310	0.0530*	
H9A	0.51590	0.13150	0.60780	0.0500*	
H9B	0.46130	0.04000	0.54380	0.0500*	
H10A	0.4614 (9)	−0.0236 (16)	0.755 (2)	0.0560*	

### Atomic displacement parameters (Å^2^)

**Table:**

	*U*^11^	*U*^22^	*U*^33^	*U*^12^	*U*^13^	*U*^23^
S1	0.0497 (3)	0.0379 (3)	0.0406 (3)	0.0112 (2)	0.0209 (2)	0.0037 (2)
O1	0.0372 (7)	0.0318 (6)	0.0488 (8)	0.0044 (5)	0.0212 (6)	−0.0030 (5)
N1	0.0403 (9)	0.0350 (8)	0.0413 (9)	0.0098 (6)	0.0161 (7)	0.0036 (6)
N2	0.0410 (9)	0.0330 (7)	0.0378 (9)	0.0080 (6)	0.0158 (7)	0.0024 (6)
N3	0.0367 (8)	0.0332 (7)	0.0393 (8)	0.0023 (6)	0.0146 (7)	−0.0007 (6)
C1	0.0325 (9)	0.0315 (8)	0.0385 (10)	0.0019 (7)	0.0107 (7)	−0.0044 (7)
C2	0.0339 (9)	0.0283 (8)	0.0418 (10)	0.0015 (7)	0.0110 (8)	−0.0017 (7)
C3	0.0353 (9)	0.0333 (9)	0.0399 (10)	−0.0033 (7)	0.0121 (7)	−0.0043 (7)
C4	0.0433 (11)	0.0426 (11)	0.0467 (11)	0.0020 (8)	0.0134 (9)	0.0004 (8)
C5	0.0510 (12)	0.0548 (12)	0.0437 (11)	−0.0029 (9)	0.0143 (9)	0.0068 (9)
C6	0.0555 (13)	0.0580 (13)	0.0411 (11)	−0.0120 (10)	0.0215 (9)	−0.0062 (9)
C7	0.0452 (11)	0.0439 (10)	0.0493 (12)	−0.0058 (8)	0.0224 (9)	−0.0119 (9)
C8	0.0361 (9)	0.0316 (9)	0.0415 (10)	−0.0068 (7)	0.0128 (8)	−0.0078 (7)
C9	0.0348 (10)	0.0339 (9)	0.0626 (13)	0.0007 (7)	0.0217 (9)	−0.0127 (8)
C10	0.0335 (14)	0.0252 (12)	0.086 (2)	0.0000	0.0227 (14)	0.0000

### Geometric parameters (Å, º)

**Table:**

S1—C1	1.6945 (19)	C5—C6	1.384 (3)
O1—C8	1.375 (2)	C6—C7	1.380 (3)
O1—C9	1.441 (2)	C7—C8	1.399 (3)
N1—C1	1.326 (2)	C9—C10	1.503 (2)
N2—N3	1.374 (2)	C2—H2	0.9500
N2—C1	1.341 (2)	C4—H4	0.9500
N3—C2	1.280 (2)	C5—H5	0.9500
N1—H1A	0.9100	C6—H6	0.9500
N1—H1B	0.9100	C7—H7	0.9500
C2—C3	1.460 (3)	C9—H9A	0.9900
N2—H2A	0.9100	C9—H9B	0.9900
C3—C4	1.391 (3)	C10—H10A	0.970 (19)
C3—C8	1.398 (3)	C10—H10A^i^	0.970 (19)
C4—C5	1.383 (3)		
			
C8—O1—C9	116.91 (14)	O1—C9—C10	107.97 (14)
N3—N2—C1	119.38 (15)	C9—C10—C9^i^	114.84 (19)
N2—N3—C2	115.31 (15)	N3—C2—H2	120.00
S1—C1—N1	122.17 (14)	C3—C2—H2	120.00
S1—C1—N2	120.20 (13)	C3—C4—H4	119.00
N1—C1—N2	117.62 (16)	C5—C4—H4	119.00
H1A—N1—H1B	119.00	C4—C5—H5	120.00
C1—N1—H1B	120.00	C6—C5—H5	120.00
C1—N1—H1A	120.00	C5—C6—H6	120.00
C1—N2—H2A	127.00	C7—C6—H6	120.00
N3—N2—H2A	113.00	C6—C7—H7	120.00
N3—C2—C3	120.78 (16)	C8—C7—H7	120.00
C4—C3—C8	118.49 (17)	O1—C9—H9A	110.00
C2—C3—C8	120.12 (16)	O1—C9—H9B	110.00
C2—C3—C4	121.38 (17)	C10—C9—H9A	110.00
C3—C4—C5	121.55 (19)	C10—C9—H9B	110.00
C4—C5—C6	119.26 (19)	H9A—C9—H9B	108.00
C5—C6—C7	120.71 (19)	C9—C10—H10A	107.0 (12)
C6—C7—C8	119.81 (19)	C9—C10—H10A^i^	112.0 (12)
O1—C8—C3	116.25 (16)	C9^i^—C10—H10A	112.0 (12)
C3—C8—C7	120.18 (17)	H10A—C10—H10A^i^	103.6 (17)
O1—C8—C7	123.57 (17)	C9^i^—C10—H10A^i^	107.0 (12)
			
C9—O1—C8—C3	−172.46 (16)	C2—C3—C8—O1	−2.2 (3)
C9—O1—C8—C7	6.8 (3)	C2—C3—C8—C7	178.46 (18)
C8—O1—C9—C10	172.29 (14)	C4—C3—C8—O1	178.61 (16)
C1—N2—N3—C2	−178.70 (16)	C4—C3—C8—C7	−0.7 (3)
N3—N2—C1—S1	177.07 (13)	C3—C4—C5—C6	0.0 (3)
N3—N2—C1—N1	−2.2 (2)	C4—C5—C6—C7	−0.3 (3)
N2—N3—C2—C3	177.81 (16)	C5—C6—C7—C8	0.1 (3)
N3—C2—C3—C4	−3.1 (3)	C6—C7—C8—O1	−178.85 (18)
N3—C2—C3—C8	177.76 (17)	C6—C7—C8—C3	0.4 (3)
C2—C3—C4—C5	−178.67 (18)	O1—C9—C10—C9^i^	−59.22 (16)
C8—C3—C4—C5	0.5 (3)		

Symmetry code: (i) −*x*+1, *y*, −*z*+3/2.

### Hydrogen-bond geometry (Å, º)

**Table:**

*D*—H···*A*	*D*—H	H···*A*	*D*···*A*	*D*—H···*A*
N1—H1*A*···N3	0.91	2.27	2.631 (2)	103
N1—H1*A*···S1^ii^	0.91	2.64	3.3393 (16)	135
N1—H1*B*···O1^iii^	0.91	2.20	3.1046 (19)	176
N2—H2*A*···S1^iv^	0.91	2.49	3.3909 (16)	171

Symmetry codes: (ii) *x*, −*y*+1, *z*−1/2; (iii) −*x*+1/2, *y*+1/2, −*z*+3/2; (iv) −*x*+1/2, −*y*+1/2, −*z*+2.
