# Supplementary figures and images for: Crystal structure of [(E)-({2-[3-(2-{(1E)-[(carbamo­thioyl­amino)­imino]­meth­yl}phen­oxy)prop­oxy]phen­yl}methyl­idene)amino]­thio­urea with an unknown solvate
Source: Acta Crystallogr E Crystallogr Commun. 2015 Jun 30;71(Pt 7):o530–1. doi: 10.1107/S2056989015012074 (PMC4518929; doi:10.1107/S2056989015012074)

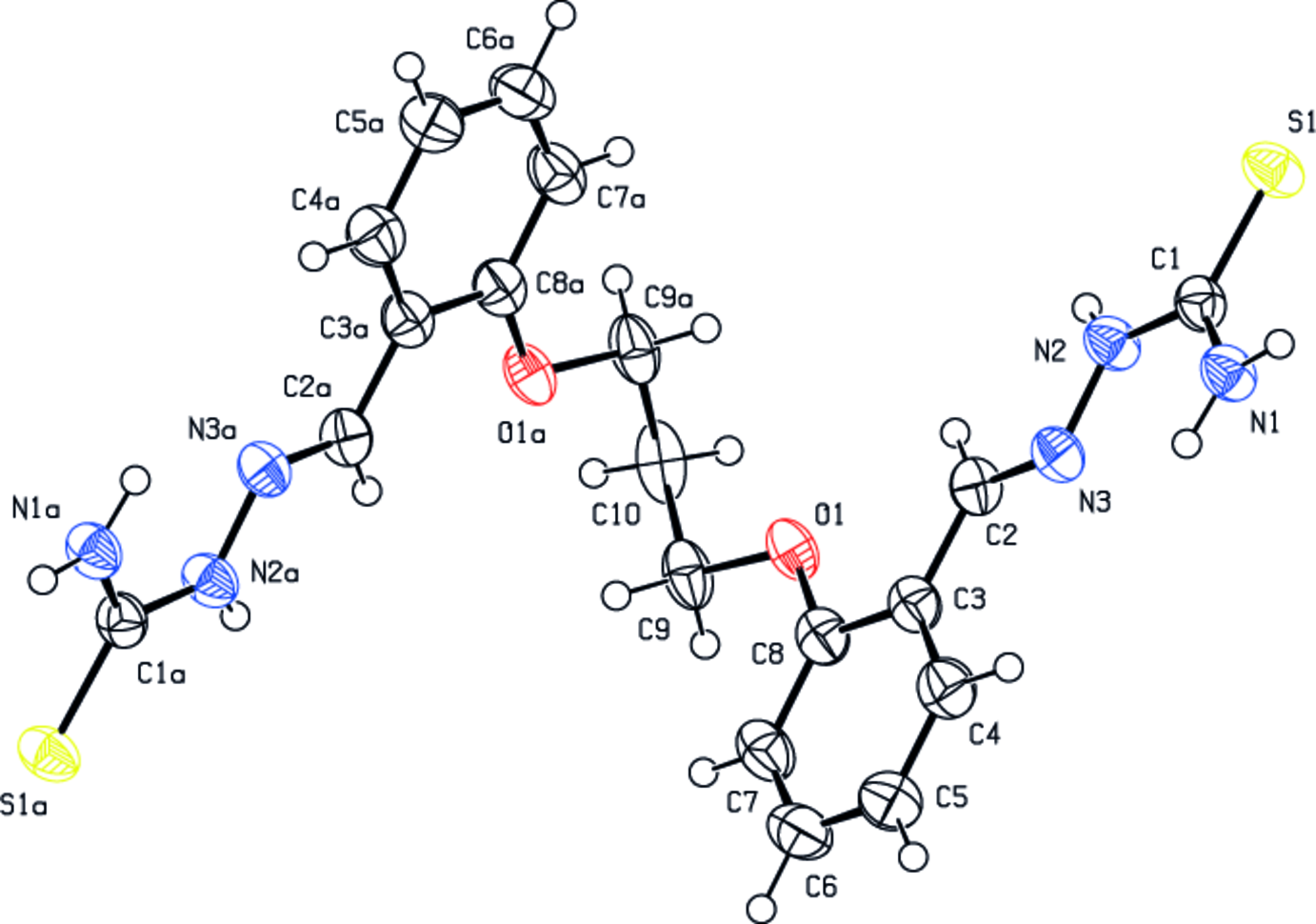

Supplement: Supplementary file 4 [file e-71-0o530-fig1.tif]

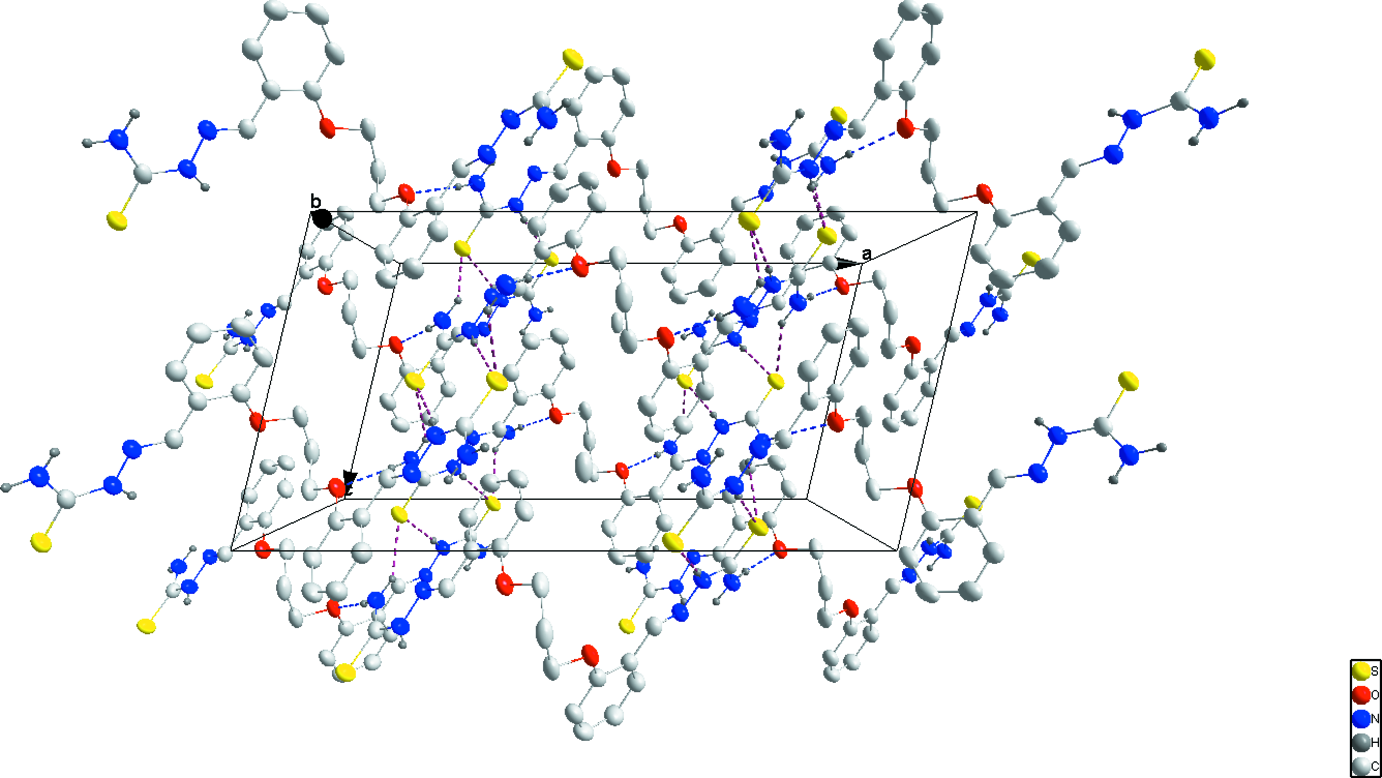

Supplement: Supplementary file 5 [file e-71-0o530-fig2.tif]

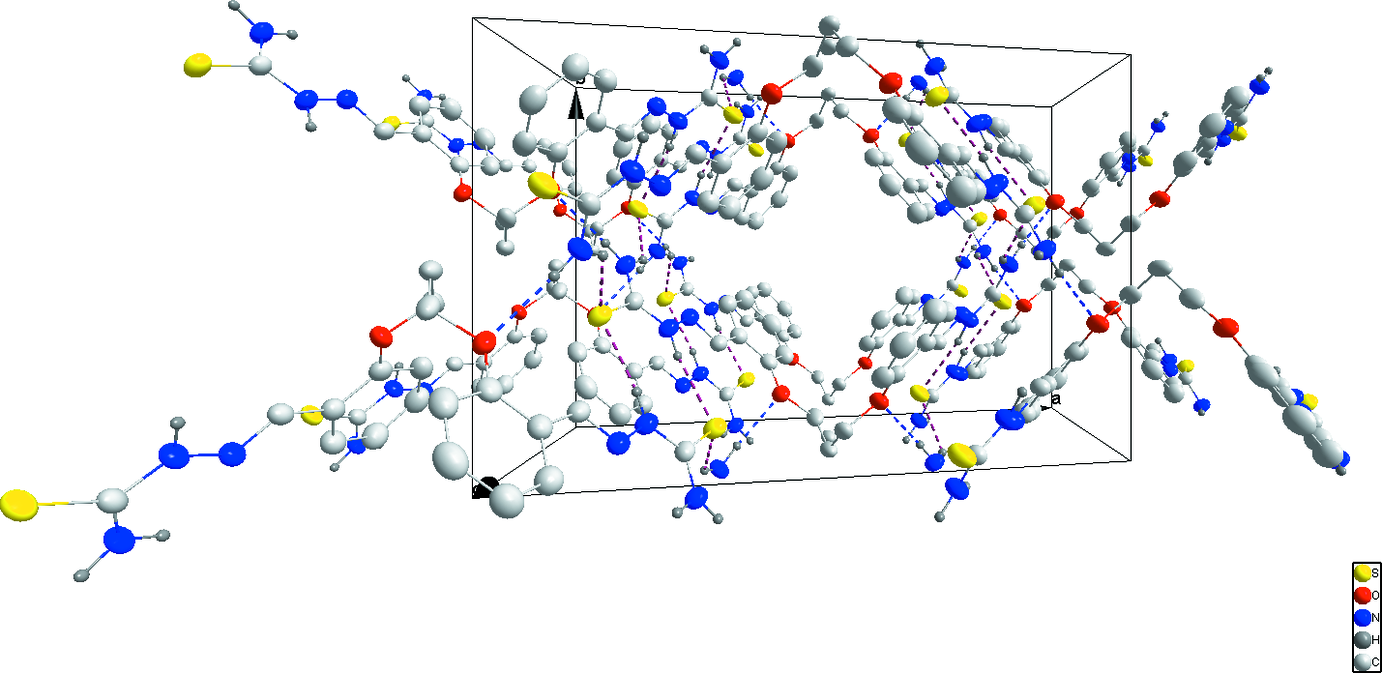

Supplement: Supplementary file 6 [file e-71-0o530-fig3.tif]
